# Is the presence of HCMV components in CNS tumors a glioma-specific phenomenon?

**DOI:** 10.1186/s12985-019-1198-5

**Published:** 2019-08-01

**Authors:** Daling Ding, Ailing Zhao, Zhi Sun, Lihua Zuo, Anhua Wu, Jianrui Sun

**Affiliations:** 1 Department of Neurosurgery, The First Affiliated Hospital of Zhengzhou University, Zhengzhou, 450052 Henan China; 2 Department of Infant Ward, Children’s Hospital Affiliated of Zhengzhou University, Zhengzhou, 450052 China; 3 Department of Pharmacy, The First Affiliated Hospital of Zhengzhou University, Zhengzhou, 450052 China; 4 Department of Neurosurgery, The First Affiliated Hospital of China Medical University, Shenyang, 110001 China

**Keywords:** Glioma, Human cytomegalovirus, Central nervous system, Immunohistochemistry

## Abstract

**Background:**

Human cytomegalovirus (HCMV) has been associated with malignant gliomas. The purpose of the present study was to investigate the presence of HCMV in common non-glial tumors of the central nervous system (CNS) and to determine whether it is a glioma-specific phenomenon.

**Methods:**

Using HCMV-specific immunohistochemical staining, HCMV proteins IE1–72 and pp65 were examined in 65 meningiomas (benign, atypical and malignant), 45 pituitary adenomas, 20 cavernous hemangiomas, and 30 metastatic carcinomas specimens. HCMV DNA was also measured in these tumor tissues and the peripheral blood from patients using nested PCR.

**Results:**

In meningioma, IE1–72 was detected in 3.1% (2/65) and pp65 was detected in 4.6% (3/65), whereas no IE1–72 and pp65 were detected in atypical and malignant meningioma. A low level of IE1–72 immunoreactivity 6.7% (2/30) was detected in metastatic carcinoma; pp65 was not detected. No HCMV components were detected in pituitary adenoma and cavernous hemangioma. The results of immunohistochemical staining were confirmed by HCMV-specific PCR. HCMV DNA was not detected in the peripheral blood of the non-glial CNS tumors patients.

**Conclusions:**

Our results demonstrate that the presence of HCMV components is not an entirely glioma-specific phenomenon, and that HCMV is present in a low percentage in some non-glioma CNS tumors. Comparing HCMV-positive non-glial CNS tumors with HCMV-positive gliomas may cast light on the mechanism and role of HCMV in CNS tumors.

**Electronic supplementary material:**

The online version of this article (10.1186/s12985-019-1198-5) contains supplementary material, which is available to authorized users.

## Background

It has been reported that viral infections may be responsible for specific human cancers worldwide [1–3]. One example is of human cytomegalovirus (HCMV), which is carried by the majority of the population worldwide. HCMV is a leading cause of opportunistic and congenital disease and is the most frequent infectious cause of developmental disorders of the CNS in humans [4, 5]. Some scholars [6–10] reported the presence of active HCMV infection in gliomas. Our previous study detected HCMV IE1 immunoreactivity in 76.1% of glioma specimens of various grades, and pp65 in 65.7%, whereas HCMV proteins and viral gene expression were not detected in control brain tissue specimens [11], a finding consistent with the results of Cobbs et al. [7] and Mitchell et al. [12]. We also observed that the presence of HCMV components does not correlate with the prognosis and other risk factors of glioma patients. Our previous study suggested that the presence and role of HCMV in gliomas may be dependent upon the specific local tumor microenvironment [11]. If a similar microenvironment exits in non-glial tumors of the CNS, then it is possible that HCMV components might also be present in these tumors, and local HCMV infection may therefore not be a glioma-specific phenomenon. The purpose of this study was to investigate the presence of HCMV components in common non-glial CNS tumors and thus to determine whether the presence of HCMV is glioma-specific.

## Methods

### Clinical samples

Tumor tissues were obtained during surgical resections at the Department of Neurosurgery of the First Affiliated Hospital of Zhengzhou University from January 2014 to December 2018. The general data of the patients, including age, sex, date of surgery, extent of resection were collected from the medical records. No patients underwent chemotherapy or radiation prior to surgical therapy. Samples for this research consisted of 65 meningiomas (benign, atypical, and malignant), 45 pituitary adenomas, 20 cavernous hemangiomas, and 30 metastatic carcinomas. The meningiomas were classified into benign (WHO grade I), 57 cases, and atypical/malignant, 8 cases. The pituitary adenomas consisted of 15 cases of growth hormone (GH) pituitary adenoma, 19 prolactin (PRL) pituitary adenomas, and 11 adrenocorticotropic hormone (ACTH) pituitary adenomas. Of these, 12 were invasive pituitary adenomas. Detailed information on the clinical characteristics of the non-glial CNS tumors patients is listed in Table 1.

**Table 1:** Clinical and molecular characteristics of the non-glial CNS tumors patients

Histology	n	Sex	Age (Years)	Extent of	resection	IE1–72	pp65	Nest-PCR
		M/F	Mean ± SD	Total removal (%)	Subtotal removal (%)	Positive of No. (%)	Positive of No. (%)	Positive of No. (%)
meningoma	65	24/41	48.76 ± 12.69	60 (92.3%)	5 (7.7%)	2 (3.1%)	3 (4.6%)	2 (3.1%)
benign	57	18/39	48.14 ± 12.84	54 (94.7%)	3 (5.3%)	2 (3.5%)	3 (5.3%)	2 (3.5%)
atypical/malignant	8	6/2	53.25 ± 11.34	6 (75.0%)	2 (25.0%)	0	0	0
pituitary adenoma	45	11/34	41.27 ± 10.44	33 (73.3%)	12 (26.7%)	0	0	0
GH	15	3/12	42.13 ± 8.29	11 (73.3%)	4 (26.7%)	0	0	0
PRL	19	2/17	42.11 ± 11.36	14 (73.7%)	5 (26.3%)	0	0	0
ACTH	11	6/5	38.64 ± 11.85	8 (72.7%)	3 (27.3%)	0	0	0
cavernous hemangioma	20	7/13	47.05 ± 11.09	19 (95%)	1 (5%)	0	0	0
metastatic carcinoma	30	18/12	56.40 ± 12.27	28 (93.3%)	2 (6.7%)	2 (6.7%)	0	2 (6.7%)

After surgical resection, samples were divided into two; one part was processed for histopathology studies and the other was transferred into cryotubes and immediately snap-frozen in liquid nitrogen for subsequent molecular analysis. The histological diagnosis was established and verified by two neuropathologists according to the 2016 WHO classification guidelines [13]. For each sample, the percentage of tumor cells was assessed on hematoxylin and eosin-stained frozen sections. Peripheral blood samples were obtained from each patients. This study was approved by the institutional review boards of the hospital and written informed consent was obtained from all patients.

### Immunohistochemistry

Tumor tissues were paraffin-embedded, and 4 μm sections were analyzed by sensitive immunostaining as described [7, 12]. Briefly, all sections were deparaffinized in xylene and rehydrated through serial dilutions of ethanol. The sections were blocked for endogenous peroxidase activity (3% H_2_O_2_, 12 min) and incubated with Fc receptor blocker (10 min, 20 °C; Innovex Biosciences, Richmond, CA, USA) before the addition of monoclonal antibodies. Sections were incubated at 4 °C overnight with either anti-IE1–72 (1:40; Abcam, Hong Kong) or anti-pp65 (clones 2 and 6, 1:50, Abcam, Hong Kong). Negative control sections not treated with primary antibody were also included. The slides were then developed with a secondary antibody (Dako Cytomation, Carpinteria, CA). Antigen-antibody complexes were visualized by streptavidin-conjugated horseradish peroxidase (Bio-Maixin, Fu Zhou, KIT-9701) and 3,3′-diaminobenzidine (DAB) as a chromogen, and counterstained with hematoxylin.

Sections were evaluated independently under light microscopy by two investigators blinded to clinical data. The evaluated proportion of positive cells was classified as 0 (0–1%), 1 (2–25%), 2 (26–50%), 3 (51–75%), or 4 (> 75%). For viral antigens, staining intensity was classified as negative, weak, moderate, or strong, with corresponding scores from 0 to 3, referring to the predominant intensity. A combined staining index was determined by multiplying the proportion by the staining intensity. A staining index > 4 was defined as tumors with widespread high IE1–72 or pp65 levels, and a staining index ≤4 as tumors with lower and/or more disseminated levels of staining.

### Nested PCR analysis

DNA was extracted from tissue or 300 μl of peripheral blood using the Wizard® Genomic DNA Purification Kit (Promega, Madison, WI, USA) according to the manufacturer’s instructions. DNA was quantitated by absorbance at 260 nm. The final DNA extracts were stored at − 80 °C until use.

The detection of HCMV in DNA samples was performed by nested PCR analysis with external and internal primers specific for the HCMV UL55 gene region. External primers (E-1/E-2) amplify a 267 bp fragment whereas internal primers (I-1/I-2) amplify a 146 bp fragment. The primer pairs used and the size of the amplified fragments are shown in Table 2. All samples were tested at least in triplicate. The first round of PCR was carried out in a 25 μl reaction containing 0.25 U of Taq polymerase, 0.5 μl of each external primer (10 mM) and 2 μl of each genomic DNA sample. For the first amplification round, after an initial denaturing step (94 °C, 5 min), 35 cycles of denaturation (94 °C, 30 s), annealing (58 °C, 30 s), extension (72 °C, 30 s) were performed, followed by a final extension step (72 °C, 6 min). Amplified products (2 μl) were then subjected a second round of PCR using the primer pair I-1/I-2; cycling parameters were denaturation (94 °C for 5 min) followed by 40 cycles of amplification (94 °C, 30 s; 59 °C, 30 s; 72 °C, 30 s) followed by a final extension step (72 °C, 5 min). Following second-round amplification, 5 μl PCR products were electrophoresed on 2.0% agarose gel, stained with ethidium bromide, and photographed on an ultraviolet light transilluminator. PCR products were extracted and subjected to direct DNA sequence analysis. During the PCR process, strict precautions were taken to avoid cross-contamination [14].

**Table 2:** Sequence of primers used in the PCR for HCMV

Primer	Sequence	Product size in base pairs (bp)
E-l	5′-TCCAACACCCACAGTACCCGT-3′	267
E-2	5′-CGGAAACGATGGTGTAGTTCG-3′	
I-1	5′-TGACGGTCA AGGATCAGTGGC-3′	146
I-2	5′-GTA AACCACATCACCCGTGGA-3′	

## Results

### Immunohistochemical detection of HCMV IE1–72 proteins

**Fig. 1:**
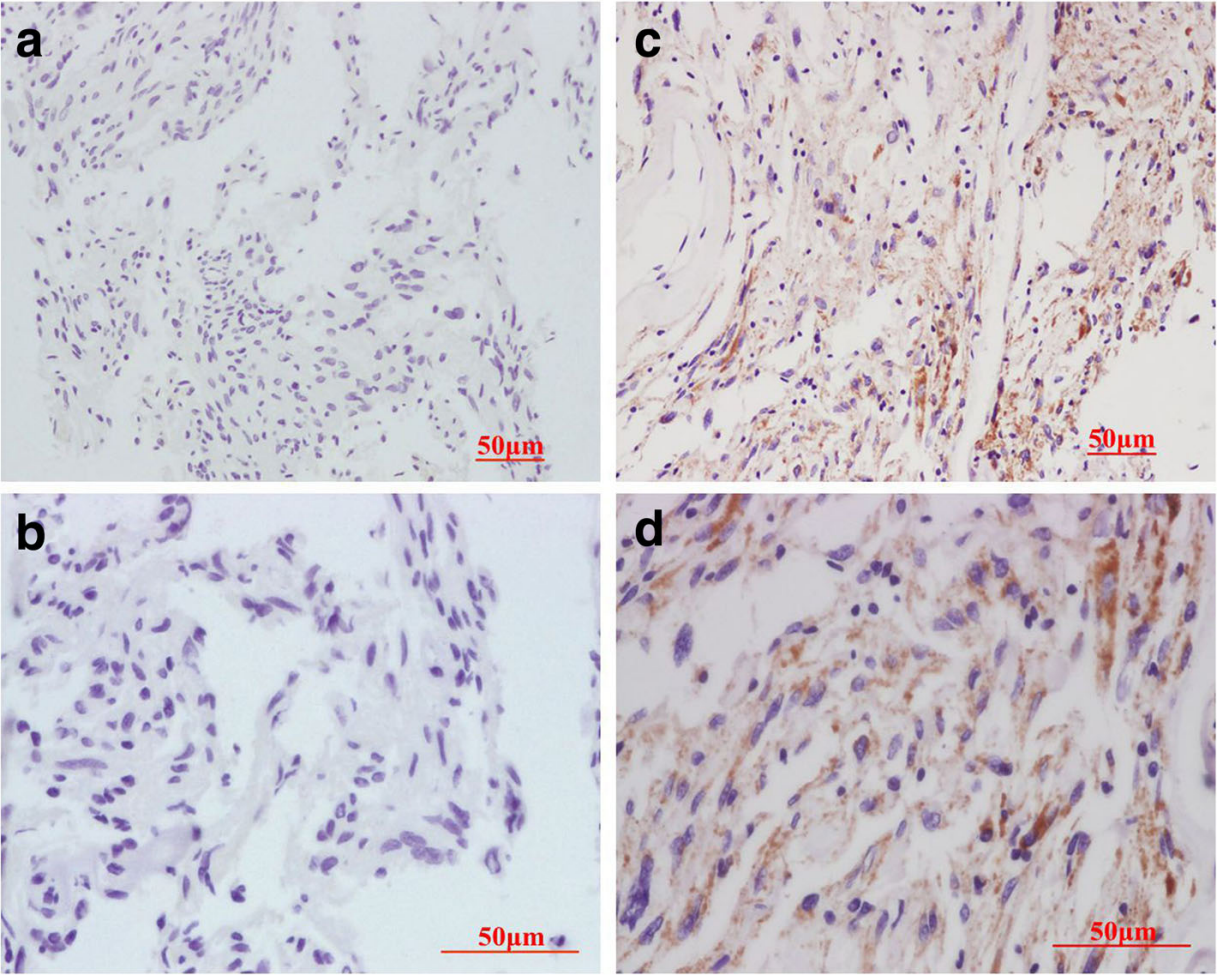
Immunohistochemical staining of tumor sections showing expression of HCMV IE1–72 in meningioma. IE1–72 immunoreactivity is observed in a benign meningioma (**c, d**) and not detected in another benign meningioma (**a, b**). **a** and **c** are low-power images; **b** and **d** are high-power images. Scale bar: 50 μm

Immunohistochemical staining was used to detect whether HCMV proteins are expressed in a series of common non-glial CNS tumors including meningioma, pituitary adenoma, cavernous hemangioma, and metastatic carcinoma. We optimized immunohistochemistry conditions for the detection of low levels of HCMV IE1–72 expression. IE1–72 expression was detected in 3.1% (2/65) of meningioma specimens, whereas no IE1–72 protein was detected in atypical/malignant meningioma (Fig. 1a and b). Only a low percentage of IE1–72 immunoreactivity (6.7%, 2/30) was detected in metastatic carcinoma, and we did not detect IE1–72 immunoreactivity in pituitary adenoma or cavernous hemangioma. In HCMV-positive tumors, IE1–72 immunoreactivity was found in the nuclei and the perinuclear cytoplasm (Fig. 1c, d and Fig. 3c, d), and blood vessel endothelial cells within tumors were also HCMV-positive.

### Immunohistochemical detection of HCMV pp65 proteins

**Fig. 2:**
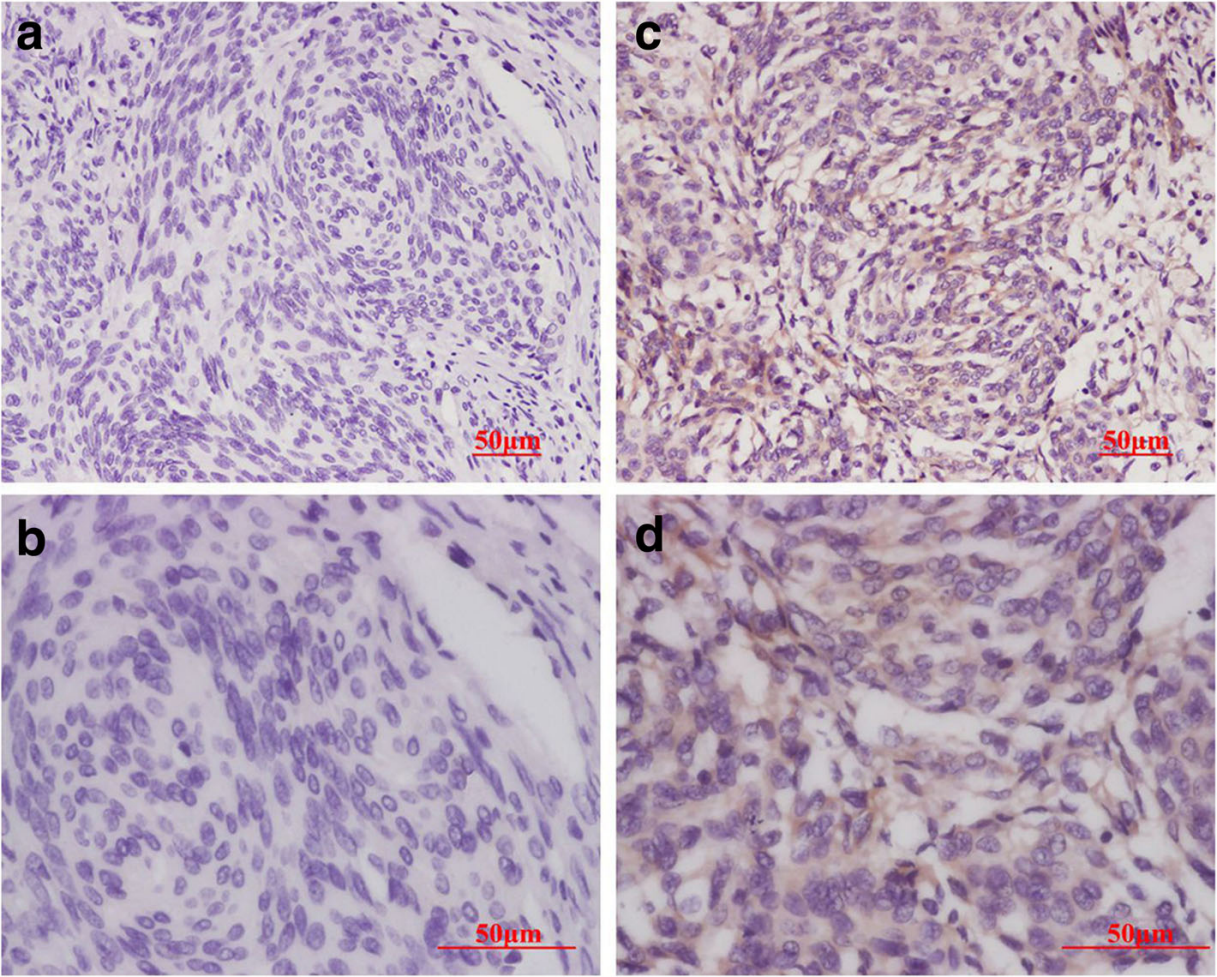
Immunohistochemical staining of HCMV pp65 in meningioma. HCMV pp65 immunoreactivity is detected in a benign meningioma (**c**, **d**) and not detected in another benign meningioma (**a, b**). **a** and **c** are low-power images; **b** and **d** are high-power images. Scale bar: 50 μm

**Fig. 3:**
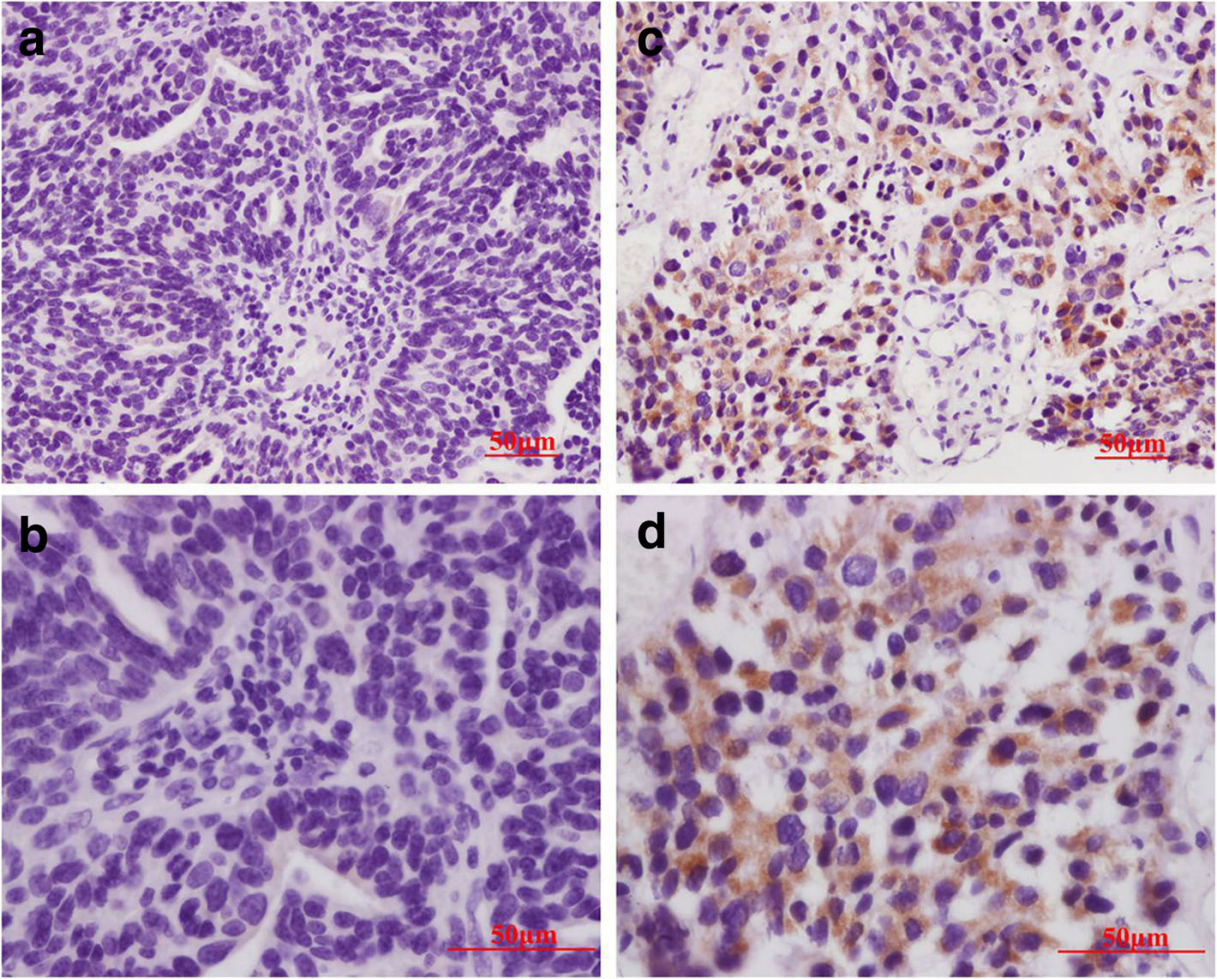
Immunohistochemical staining of HCMV in metastatic carcinoma. HCMV IE1–72 immunoreactivity is observed in a metastatic carcinoma (**c, d**), no pp65 are detected in another metastatic carcinoma tissues (**a**, **b**). **a** and **c** are low-power images; **b** and **d** are high-power images. Scale bar: 50 μm

To confirm HCMV antigen expression in these samples, HCMV-related protein pp65 was also investigated. We detected the pp65 tegument protein in 4.6% (3/65) of meningioma specimens (Fig. 2c and d), among the 3 pp65 positive specimens, two specimen were also positive for IE1–72 as indicated above; no pp65 expression was detected in atypical/malignant meningioma. HCMV pp65 protein was not detected in pituitary adenoma, cavernous hemangioma, or metastatic carcinoma. Meanwhile, no immunohistochemical staining was detected in negative control sections (Fig. 2a,b and Fig. 3a,b). Our results indicate that HCMV immunoreactivity is only present in a low percentage of common non-glial CNS tumors (Table 1).

### Nested PCR analysis of HCMV DNA

**Fig. 4:**
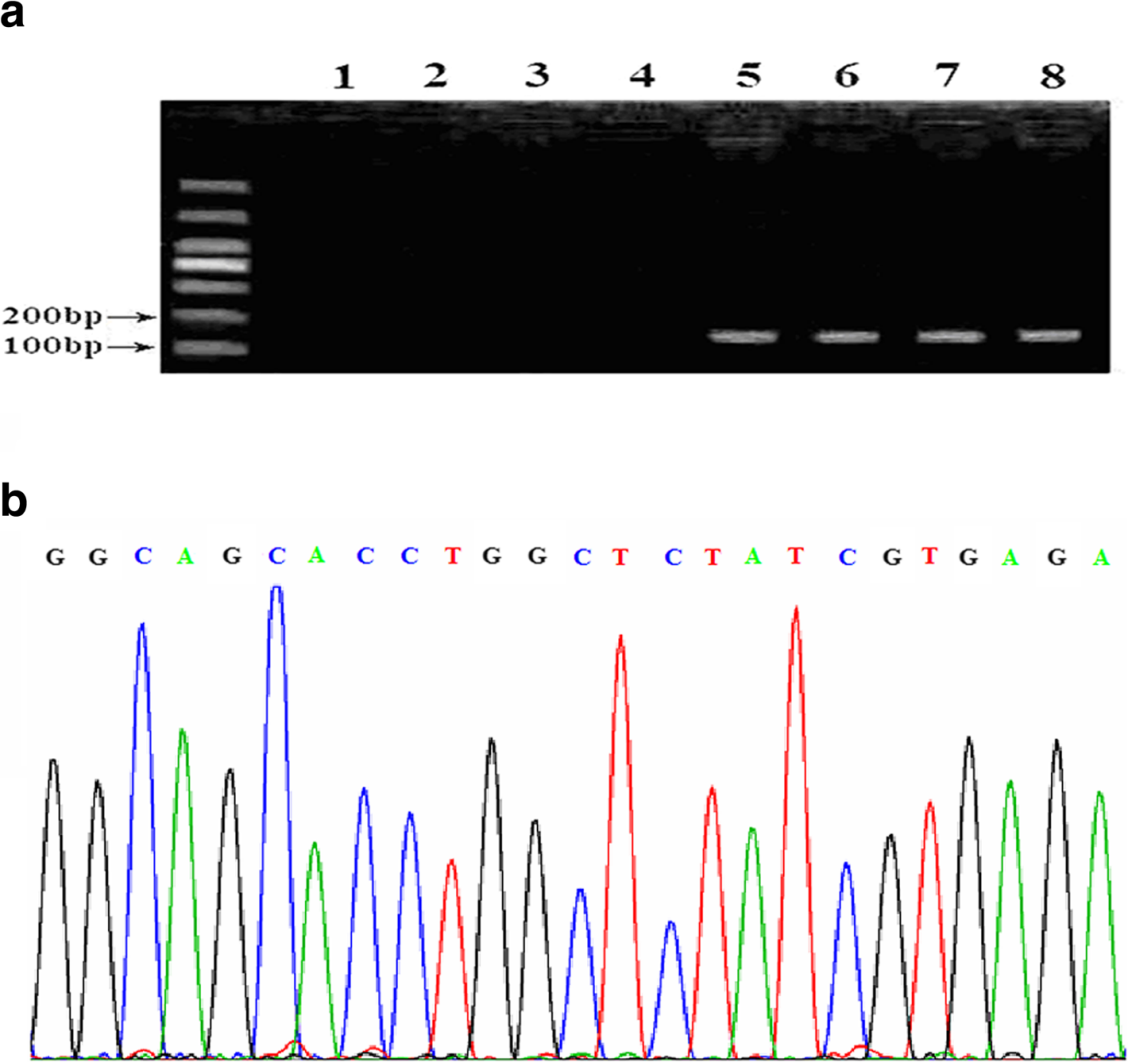
**a** Nested PCR analysis of viral DNA from clinical samples. Nested PCR demonstrates the presence of HCMV UL55 DNA sequences (146 bp) in tumor tissues of meningioma and metastatic carcinoma samples, but not in pituitary adenoma and cavernous hemangioma samples. Clinical samples: lanes 1,2, pituitary adenoma; lanes 3,4, cavernous hemangioma; lanes 5,6, metastatic carcinoma; lanes 7,8, meningioma. Positive samples in lanes 5–8 are the same samples positive for antigen reactivity. **b** Sequencing of the 146 bp PCR product

To confirm the presence of HCMV in these tumors we performed nested PCR to detect HCMV UL55 sequences in total DNA extracted from all studied tumor samples and control tissues. Samples were considered HCMV-positive when a band of 146 bp was present. HCMV sequences were only detected in the four antigen-positive samples detected earlier, specifically two benign meningiomas and two metastatic tumors. This confirmed in all cases that these products were HCMV sequences. No HCMV DNA was detected in pituitary adenoma or cavernous hemangioma tissues (Fig. 4a and b). Unfortunately, HCMV DNA was not detected in the peripheral blood of the non-glial CNS tumors patients (Additional file 1: Figure S1).

## Discussion

It is now well accepted that ~ 20% of the global cancer burden can be associated with infectious agents including viruses, bacteria, and parasites [15]. Recently, studies showed that HCMV is likely to play a role in malignancies. HCMV is a widespread betaherpesvirus carried by 70–90% of the world population, and persists for the lifetime of its host after primary infection. In healthy persons, HCMV infection is generally asymptomatic [9, 16, 17]. Cobbs et al. [7] first reported that HCMV nucleic acids and proteins are present in a high percentage of low-and high-grade malignant gliomas, and others have also detected HCMV components in glioma tissues. In our previous study we detected IE1–72 immunoreactivity in 76.1% of malignant glioma specimens of various grades, and pp65 in 65.7%; no IE1–72- or pp65-positive cells were detected in control brain tissue specimens [11], a finding consistent with the results of Cobbs et al. [7] and Mitchell et al. [12]. However, in other studies [18–22], no evidence of a direct association between malignancies and HCMV infection was found, and the association of HCMV with malignancies is still controversial, with conflicting reports in the literature [7–9, 16, 18–32]. Whether HCMV plays a role in the tumor progression, or whether tumor growth simply provides a supportive environment for local reactivation and propagation of the virus, is also generally unclear.

The role of HCMV infection on the development and progression of tumor is uncertain. There are three possibilities. First, HCMV infection might promote tumor cell proliferation and invasion. Thus, HCMV-positive tumor cells could have a survival advantage in tumor tissues and their abundance might increase as tumor progression. This is not supported by our previous report and by the current findings. Second, HCMV infection might inhibit tumor cell proliferation and invasion. This would lead to a decrease and loss of HCMV-positive cells in tumor tissues, and this is not supported by the evidence. Finally, HCMV has no substantial effect on tumor cell proliferation and is merely a bystander. However, this interpretation also runs counter to several experimental results [6, 7, 12, 16, 27, 33]. In our previous study we found that the relationship between HCMV-infection and glioma proliferation/invasion is complex and may be determined by the specific local microenvironment of different glioma tissues. Importantly, if the majority of gliomas are found to display reactivation and replication of latent HCMV, then antiviral therapy against HCMV would warrant consideration as a possible adjunct to conventional glioma therapeutic intervention. It is therefore important to determine if HCMV expression is also present in non-glial tumors, and whether anti-HCMV therapy might also be envisaged in other types of CNS tumor.

In this study we investigated the presence of HCMV in non-glial CNS tumors in a relatively large series of samples. Unexpectedly, HCMV immunoreactivity was detected only in a low percentage of non-glial CNS tumors. Owing to the low rate of HCMV-positive samples, statistical analysis of the potential relationship between the existence of HCMV components and other parameters including gender, age, and tumor grade and site, was not performed. However, our previous study on gliomas indicated that these factors are unrelated to HCMV positivity.

The presence of HCMV in gliomas may reflect the specific local tumor microenvironment, and gliomas and metastatic carcinomas share similar features of high proliferation rates and invasion. Surprisingly, however, HCMV was only detected in a low percentage in brain metastatic carcinomas. This could indicate that the cellular microenvironment differs significantly between gliomas and metastatic carcinomas; further analysis of the microenvironments and tumor cell characteristics in HCMV-positive metastatic carcinomas and gliomas may cast light on the presence and role of HCMV in different tumor types. In meningioma HCMV expression was detected in only a small percentage of samples, and HCMV was not detected in atypical/malignant meningioma. These observations provide no evidence that increased frequency and intensity of HCMV expression accompanies malignant progression of non-glial CNS tumors, suggesting that HCMV infection may not be related to malignant transformation.

However, our study has several limitations, notably that we only analyzed a small number of malignant meningioma samples, and this could potentially explain our failure to detect HCMV positivity in malignant meningioma. However, in the overall series of samples, including both benign and malignant tumors, only a low rate of HCMV positivity was found in meningioma, and this was far less than the HMCV-positive rate previously reported in glioma [11]. Second, our study was based on retrospective analysis, systematic bias may be influence the accuracy of the results. Last but not least, although the current study enrolled a relatively large sample size in non-glioma CNS tumors, it is a single-cencer study, further multi-center prospective studies are necessary to corroborate our findings.

Different characteristics of the tumor cells and microenvironment may underlie the different rates of HCMV detection. In light of similar researches such as Bianchi E et al. [34], the results highlight that the histological grade and degree of malignancy of CNS tumours were not commonly a discriminating factor when evaluating the presence of the virus. It has been reported that HCMV infection and amplification are related to the expression of epidermal growth factor receptor (EGFR), platelet-derived growth factor receptor (PDGFR), and Toll-like receptors (TLRs), as well as integrin [35–40]. It was speculated that the high expression of the above factors in glioma or other tumors may be the cause of the positive staining for HCMV, but this remains to be confirmed by further experiments.

In glioma, HCMV IE1 immunoreactivity was localized in the nuclei and the perinuclear cytoplasm, but not in areas of adjacent normal brain tissue [7, 12, 16, 27, 33]. In addition, Scheurer et al. [16] and Rahbar et al. [27] found that blood vessels within tumors were positive for HCMV proteins, and Scheurer et al. reported that the endothelial cells of blood vessels in the tumor were more immunoreactive for HCMV in anaplastic and low-grade tumors than in the glioblastoma multiforme samples. In the current report, and in previous studies on gliomas, the staining type was comparable between different tumor types, with both perinuclear cytoplasmic and nuclear staining for HCMV antigens in tumor cells, and blood vessel endothelial cells within tumors were also HCMV-positive. However, interestingly, although HCMV was present in the vascular endothelium within tumors, it was not associated with vascular malformation (negative in cavernous hemangioma samples). These results indicated that HCMV positivity is not specific for particular cell types within tumors, and may be related to the specific local microenvironment, or be a relative tumor type-specific phenomenon.

## Conclusions

Our result demonstrate that HCMV protein and nucleic acid are present in a low percentage of non-glial CNS tumors, and therefore that HCMV positivity is not glioma-specific. However, the rate of HCMV positivity in non-glial tumors was much less than in glioma samples, indicative of a unique relationship between glioma and HCMV. Further in-depth study on this relationship may provide a new framework for understanding the origin, progression, and treatment of glioma. It remains an open question whether specific therapy against HCMV might inhibit the growth of HCMV-positive tumor cells and prolong the survival of patients with HCMV-positive tumors. Future work towards a better understanding of the mechanism underlying HCMV positivity of tumor tissues will need to focus on finding related factors associated with HCMV positivity, on the precise role of HCMV infection, and on whether expression of the HCMV genome affects the biologic behavior of these HCMV-positive tumors.

## Additional file

Additional file 1: **Table S1.** Expression of HCMV proteins and DNA in glioma and non glial tumors of CNS. **Figure S1**. Nested PCR analysis of HCMV DNA from peripheral blood samples. No HCMV DNA was detected in benign meningioma (lane 1), malignant meningioma(lane 2), PRL pituitary adenoma(lane 3), GH pituitary adenoma(lane 4), ACTH pituitary adenoma(lane 5), cavernous hemangioma(lane 6) and metastatic carcinoma samples(lane 7) (DOCX 41 kb)

## Data Availability

That the data will not be shared because the methods used for the experiment are standard and widely used in the scientific community.

## Abbreviations

ACTH: Adrenocorticotropic hormone
CNS: Central nervous system
DAB: 3,3′-diaminobenzidine
EGFR: Epidermal growth factor receptor
GH: Growth hormone
HCMV: Human cytomegalovirus
PDGFR: Platelet-derived growth factor receptor
PRL: prolactin
TLRs: Toll-like receptors
