# Supplementary material for: Is the presence of HCMV components in CNS tumors a glioma-specific phenomenon?
Source: Virol J. 2019 Aug 1;16:96. doi: 10.1186/s12985-019-1198-5 (PMC6670132; doi:10.1186/s12985-019-1198-5)
Supplement: Supplementary file 1 — Table S1. Expression of HCMV proteins and DNA in glioma and non glial tumors of CNS. Figure S1. Nested PCR analysis of HCMV DNA from peripheral blood samples. No HCMV DNA was detected in benign meningioma (lane 1), malignant meningioma(lane 2), PRL pituitary adenoma(lane 3), GH pituitary adenoma(lane 4), ACTH pituitary adenoma(lane 5), cavernous hemangioma(lane 6) and metastatic carcinoma samples(lane 7) (DOCX 41 kb) [file 12985_2019_1198_MOESM1_ESM.docx]

**Additional file 1: Table S1** Expression of HCMV proteins and DNA in glioma and non glial tumors of CNS

| Authors Year | | HCMV proteins | in glioma | HCMV-DNA  in glioma (%) | Meningioma  (%) | Metastatic  carcinoma |
| --- | --- | --- | --- | --- | --- | --- |
|  |  | IE1-72/IE-86 (%) | pp65(%) |  |  |  |
| Han et al | 2017 | 117/150(78%) | 99/150(66%) | 48/150(32%) |  |  |
| Yang et al | 2017 | 9/116(7.8%) |  |  |  |  |
| Stangherlin et al | 2016 | 30/52(57%) |  | 38/52(73%) |  |  |
| Xing et al | 2016 |  | 52/79(65.8%) | 43/79(54.4%) |  |  |
| Bianchi et al | 2015 | 38/66(57.6%) |  | 0 | 17/20(85%) | 4/5(80%) |
| Hung et al | 2015 | 60/60(100%) |  |  |  |  |
| Shamran et al | 2015 | 45/50(90%) | 38/50(76%) |  | 0/30(0%) |  |
| Ding et al | 2014/  present study | 51/67(76.1%) | 44/67(65.7%) | 35/67(52.2%) | 2/65(3.1%) | 2/30(6.7%) |
| dos Santos et al | 2014 |  |  | 21/22(95%) |  |  |
| Libard et al | 2014 |  | 197/219(90.0%) |  | 132/152(86.8%) |  |
| Ahani et al | 2013 |  |  | 12/16(75%) |  |  |
| Matlaf et al | 2013 |  |  | 10/15(66.7%) |  |  |
| Rahbar et al | 2013 | 79/80(99%) |  |  |  |  |
| Bhattacharjee et al | 2012 | 9/12(75%) | 9/12(75%) |  |  |  |
| Lucas et al | 2011 | 8/49(16%) | 25/49(51%) |  |  |  |
| Scheurer et al | 2008 | 44/50(88%) |  |  |  |  |
| Mitchell et al | 2007 | 42/45(93%) | 30/33(91%) | 16/20(80%) |  |  |
| Sabatier et al | 2005 | 9/81(11.1%) |  |  |  |  |
| Cobbs et al | 2002 | 27/27(100%) | 10/10(100%) |  | 0/9(0%) |  |


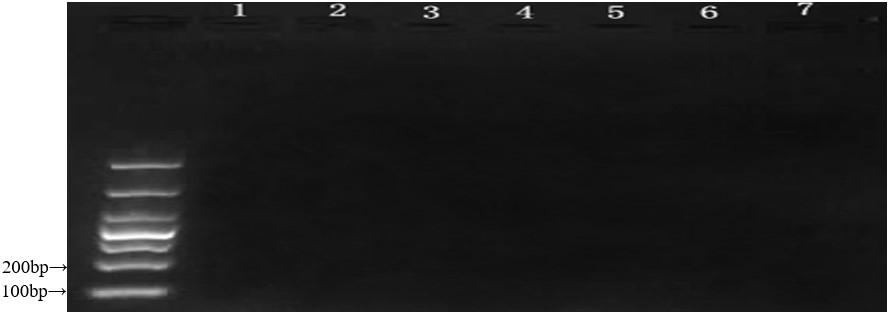


**Figure S1** Nested PCR analysis of HCMV DNA from peripheral blood samples. No HCMV DNA was detected in benign meningioma (lane 1), malignant meningioma(lane 2), PRL pituitary adenoma(lane 3), GH pituitary adenoma(lane 4), ACTH pituitary adenoma(lane 5), cavernous hemangioma(lane 6) and metastatic carcinoma samples(lane 7).
